# Dose-response assessment of *Bacillus anthracis* surrogate spore inactivation on different surfaces and environmental conditions using pH-adjusted peracetic acid

**DOI:** 10.1128/aem.02400-25

**Published:** 2026-05-19

**Authors:** Ehsan Gazi, Christine O’Sullivan, Janine Jordan, Elliot Hocknull, Debbie Padgen

**Affiliations:** 1 Dstl Porton Down 502513 , Salisbury, United Kingdom; 2 Silsoe Spray Applications Unit, Ltd. 538323, Bedford, United Kingdom; Centers for Disease Control and Prevention, Atlanta, Georgia, USA

**Keywords:** spores, dose response, environmental decontamination, *Bacillus anthracis*, biocides, remediation

## Abstract

A deliberate release of environmentally persistent *Bacillus anthracis* spores in an urban setting could cause severe public health, economic, and environmental disruption. Rapid, scalable decontamination methods are essential for effective recovery and minimizing long-term impacts. This requires understanding how decontaminant dose, surface type, and environmental conditions influence spore inactivation. The present study evaluates bespoke peracetic acid (PAA) formulations for inactivating *Bacillus thuringiensis* HD-1 cry- spores, a surrogate for *B. anthracis*. Urban surfaces nebulized with *B. thuringiensis* spores (7 Log_10_ CFU·cm⁻²) were treated with PAA (3.7% wt/wt) or PAA plus rheology modifier (RM, 4.9% wt/wt), both adjusted to pH 6 for material compatibility, and applied at ~500–2,000 L·ha⁻¹. RM was added to retain the decontaminant for longer on the surface of porous materials. Samples were incubated for 2 h under “mild” (7°C, 94% relative humidity [RH]) or “warm” (28°C, 32% RH) conditions. PAA applied at 76 kg·ha⁻¹ (delivered using 2,046 ± 43 L·ha⁻¹ [0.2 L·m⁻²]) achieved ≥5 log_10_ reduction within 2 h on porous (wood) and non-porous (steel) surfaces. At the statistically same application of 2,040 ± 42 L·ha⁻¹ (*P* = 0.69), PAA + RM showed slightly lower efficacy (≥4.6 Log_10_) despite its higher dose (98 kg·ha⁻¹). Also, a general trend of greater sporicidal efficacy was observed under warm conditions for a given surface, dose, and formulation. These data suggest a role of diffusion-dominated kinetics influencing sporicidal efficacy, where temperature and formulation viscosity can affect the level of spore inactivation. Suspension tests indicated that the virulent *B. anthracis* Ames strain was more susceptible to PAA than the surrogate. Finally, dose-response comparisons showed that PAA achieved sporicidal performance statistically equivalent to formaldehyde.

**IMPORTANCE:**

The UK Biological Security Strategy (Cabinet Office, 2023) recognizes the need for an effective response through a pre-developed, well-validated technical strategy to remediate a scene or area that has been contaminated by hazardous biological material. Although standardized American Society for Testing and Materials (ASTM) and British Standards adopted from European Norms (BS EN) methods may help initial decontaminant selection, significant information remains for remediation planners on how applied doses of decontaminants (volume and concentration) interact with different surface types and environmental conditions to influence spore reductions. By linking these parameters, this work provides critical information toward developing actionable guidance for effective and efficient incident-scale remediation. These findings are pivotal for remediation planners, who must determine the precise decontaminant quantities required, account for associated on-site storage needs, and ensure correct equipment specifications for optimized application. Additionally, although formaldehyde solution is well-proven for *B. anthracis* inactivation in the open environment, our dose-response comparison with PAA-based decontaminant provides evidence toward establishing the latter as a viable, non-carcinogenic, and safer alternative for large-scale environmental decontamination. Further exploitability of the *B. thuringiensis*-based PAA dose-response data was demonstrated by validation with virulent strains of *B. anthracis*, where it was found that surrogate-based decontamination protocols are likely to be effective against virulent *B. anthracis*.

## INTRODUCTION

The potential public health, economic, and environmental consequences of the deliberate release of hazardous and environmentally persistent *Bacillus anthracis* spores (causative agent of anthrax) into the urban environment are enormous (1). Beyond the immediate challenges of managing the consequences of such an event, technical options that can be deployed rapidly to decontaminate the affected urban areas must exist on a scale that would allow effective recovery.

The treatment of contaminated surfaces with liquid decontaminant, using spray application, represents the most likely method of remediating large outdoor areas. However, it is important that processes are optimized to reduce the volume such that it provides the target level of hazard reduction, while minimizing surface run-off and associated contamination spread. This can only be achieved through low-volume approaches that also provide logistical benefits, such as reducing the burden associated with decontaminant transport, storage, and equipment requirements.

Toward that aim, we previously reported dose-response studies showing that liquid formaldehyde spray applications reduced *Bacillus thuringiensis cry‐* spore contamination (a simulant for *B. anthracis*) on various surfaces. As little as ~1,200  L.ha^−1^ (120 mL.m^2^) achieved a 5.3 Log_10_ CFU.cm^−^² reduction on nonporous steel, while ~3,000–3,500 L.ha^−1^ (300–350 mL.m^−2^) were needed for a 4.8–6.0 Log_10_ CFU.cm^−^² reduction on porous wood (2). We subsequently validated the real-world applicability of several spray systems (such as a boom sprayer mounted onto a quad bike or mobile elevated work platforms) for delivering these target doses across a range of structures in a representative urban street (house roof, external walls, and vegetation) (3).

While formaldehyde is an effective biocide, it poses health risks due to its carcinogenicity, toxicity, and corrosive properties (4, 5). These present both a contact and vapor hazard, as well as negatively impact ecosystems. Therefore, it is preferable to develop methods that either minimize the use of formaldehyde or eliminate it entirely by employing alternative, safer decontaminants.

To this end, the present work reports dose-response data for a bespoke formulation of peracetic acid (PAA) to inactivate the spores of *B. thuringiensis cry-* on representative urban surfaces. PAA is non-carcinogenic and produces non-persistent breakdown products of acetic acid and hydrogen peroxide (National Research Council [US] Committee on Acute Exposure Guideline Levels) (6). It is readily available at large scales and listed by the US Environmental Protection Agency (EPA) as a proven sterilizer for spores (7).

PAA is an oxidizing agent, where this chemical class causes damage to the external layers of the spore, particularly the inner membrane, leading to its rupture (8, 9), but this does not involve oxidation of unsaturated fatty acids (10). Earlier research also showed that PAA-mediated inactivation neither involved DNA damage (11, 12) nor required the release of dipicolinic acid (DPA) (12).

Suspension tests using PAA concentrations of 0.1% wt/wt (pH 2.6) resulted in 6 Log_10_ CFU.mL^−1^ reduction in *B. atrophaeus,* with a 10-min contact time (13). Using the same PAA concentration, suspension test data from a study by Celebei et al. (14) demonstrated ≥5 Log_10_ CFU.mL^−1^ reduction in *B. anthracis* Sterne spores in the presence of an organic load (0.3% wt/wt BSA), a contact time of 30 min, and incubation temperatures of 4°C, 20°C, or 37°C. These suspension tests provide an initial indication of decontaminant efficacy under fully saturated (idealized) conditions, which can be used to screen relative differences in performance between formulations. However, it does not consider the complex interplay between decontaminant chemistry and its application method, the effects of environmental conditions, surface porosity and surface chemistry, spore deposition (aggregates vs. dispersed spores), and the effects of background flora, which together determine Log_10_ reduction under real-world conditions. Standardized American Society for Testing and Materials (ASTM) and British Standards adopted from European Norms (BS EN)-based disinfectant testing for spores on surfaces is available (15, 16), but these are limited in the parameters they test (non-porous surfaces only, humidity is not controlled, and unrealistic methods of disinfectant application), which raises challenges in translating results to the field.

Issues related to field translation of even laboratory soil microcosm data have been previously documented by Celebi et al. (14). That study reported no detectable spores when soil spiked in the laboratory with 4 Log_10_ *B. anthracis* Sterne spores per gram were treated with germinant and then 0.5% wt/vol PAA. However, application of this treatment regime using backpack sprayers to a *B. anthracis* contaminated site in Turkey resulted in no significant reduction in soil.

Other researchers have reported Log_10_ reductions of *B. anthracis* Ames spores on different surfaces treated with commercial PAA formulations by spray application (17). The results showed consistently high levels of spore inactivation on non-porous surfaces, but variable results on porous surfaces (brick and paving slab), depending on the decontamination chemistry. Although contact times and number of sprays were reported, this work did not quantify the applied dose and was performed in a limited range of environmental conditions (20°C–25°C and ≤70% relative humidity).

The current study advances prior work by quantifying how applied doses of PAA-based decontaminants interact with surface type and environmental conditions to influence Log_10_ reductions of *B. thuringiensis* cry- spores. Most of our assessments involved using an agricultural track-sprayer system to deliver real-world, low-volume applications, since the spray droplet-surface interactions (3) can also affect sporicidal efficacy. These findings are pivotal for remediation planners, who must determine the precise decontaminant quantities required, account for associated on-site storage needs, and ensure correct equipment specifications for optimized application.

In this work, two PAA-based decontaminants were developed (named Formulations 1 and 2) and compared for their biocidal efficacy. Both were adjusted to weakly acidic pH using sodium hydroxide, since without this adjustment, PAA is corrosive to some metals, rubber, and plastics that comprise the agricultural sprayer equipment used to apply them (18).

The formulations also contained chemical adjuvants to maximize the contact time between the solution and the spores at the material surface, as well as to improve spray delivery. These adjuvants were added at low concentrations into the formulation (≤0.3% wt/wt). To this end, both formulations contained a non-ionic ethylene oxide and propylene oxide block copolymer surfactant blend (Synperonic PE L62:64) as the wetting agent to improve surface coverage.

Formulation 2 contained additional components to the surfactant. Kelzan AP-AS (a xanthan gum) was used as a rheology modifier to improve solution retention at the surface of porous materials, while also being stable in acidic environments. Note that the preservative Proxel GXL was included in the Kelzan product for chemical storage.

Formulation 2 also contained a polydimethylsiloxane-based antifoam called Silcolapse 426R, which was necessary to reduce foaming. Foaming occurs due to the evolution of oxygen during pH adjustment of PAA, and when it is in the presence of the surfactant. This foam can depressurize spray lines, resulting in inconsistent flow rates and dose applications. In Formulation 1, the foam dissipated adequately once pH equilibrium was achieved, whereas in Formulation 2, the foam persisted due to the additional presence of Kelzan but collapsed on the addition of the antifoaming agent.

We report the applied formulation dose in L·ha⁻¹ (liters per 10,000 m²) to enable comparison with spore inactivation. This unit reflects the net sporicidal effect arising from the formulation components and their interactions with surfaces under the tested environmental conditions. In addition, we quantify the dose of PAA in kg·ha⁻¹ to establish its application rate within this context, since PAA is the primary biocide in the formulation. Moreover, we report suspension test data demonstrating that pH adjustment of PAA formulation with sodium hydroxide did not significantly affect spore viability and was not itself a biocide in the formulation.

## MATERIALS AND METHODS

### Decontaminant formulations and PAA concentration measurements

The compositions of Formulation 1 and 2 are shown in Table 1, where the “F” designation refers to a master formulation database held by Dstl.

**TABLE 1:** Formulation compositions

Formulation	Addition order	Supplier	% wt/wt
1 (F004a)	Deionised water	VWR, UK	11.50
	Sodium hydroxide (8% wt/wt)	Fisher Scientific, UK	55.00
	Peracetic acid (15% wt/wt)	Airedale Chemical, UK	33.30
	Synperonic PE L62/L64 (1:1)	Croda, UK	0.20
2 (F012a)	Sodium hydroxide (8% wt/wt)	Fisher Scientific, UK	46.50
	Kelzan AP-AS (1.5% wt/wt) with 0.1% Proxel GXL in deionized water	Prepared by Battelle UK (Kelzan supplied by CP Kelco, Proxel LanXess)	19.90
	Peracetic acid (15% wt/wt)	Airedale Chemical, UK	33.30
	Synperonic PE L62/L64 (1:1)	Croda, UK	0.20
	Silcolapse	Elkem, UK	0.10

The addition of sodium hydroxide adjusted the formulations to an initial pH of 6.7 ± 0.2 and 6.1 ± 0.1 for Formulations 1 and 2, respectively. Although the nominal final concentration of PAA was 5% wt/wt upon preparation, the accurate PAA concentration was confirmed with potentiometric titration using an autotitrator (Metrohm Titrando, Switzerland). This was operated using the Tiamo v.2.4 software, following the application note (19). A sample mass of 2 g was used when determining the accurate concentration of PAA formulations. Note that the samples were diluted in 10 mL of deionised water to provide a suitable volume for the pH probe, while not over-diluting the sample for analysis. The sample was stirred and titrated against 0.5 M sodium hydroxide (NaOH) (Sigma Aldrich, UK, product code S5881) to produce a graph of electrode potential pH (E[pH]) against volume of titrant V(mL). The slope on this graph was a function of the acetic acid (AcOH) and PAA concentrations of the sample, where their respective equivalence points (EQP1 and EQP2) were determined by the first derivative of the “shoulders” in this slope (change in pH/change in volume). The volume difference (mL) between EQP1 (AcOH)) and EQP2 (PAA) of the titrant and its molar concentration (0.5 mol/L NaOH) was used to calculate *Q* in equation 1 (the amount of NaOH added in mmol). In this equation, *C* is a constant (7.605 = Molar mass of PAA [76.05 g.mol^−1^/10]) and *m* is the sample mass (g).


(1) $$Concentration\;of\;PAA\left(\%\right)=\frac{Q\;\times C}{m}$$

Experiments comparing the efficacy of PAA with formaldehyde used (i) a 3.0% wt/wt formaldehyde formulation (Sigma Aldrich, UK) in SDW with 0.2% wt/wt Synperonic PE L62/L64 (1:1); (ii) a pH 6.02 PAA formulation comprising 3.0% wt/wt PAA (nominal dilution), 2.6% wt/wt sodium hydroxide, and 0.2% wt/wt Synperonic PE L62/L64 (1:1) in SDW.

### Spore production

A loop of the respective *Bacillus* culture stored frozen at −70°C (*B. thuringiensis* HD-1 cry- and *B. anthracis* Ames) was streaked onto plates of Tryptone Soya Agar (TSA, for *B. thuringiensis* cry-) or Luria Bertani (L-agar, for *B. anthracis* Ames) and then incubated overnight at 37°C to isolate single colonies. Three single colonies from each plate were then picked and suspended into 100 mL of pre-warmed Luria Bertani broth (L-broth) in 500 mL Erlenmeyer flasks (Product code 431145, Corning, UK). The flasks were placed on an orbital shaker (New Brunswick Scientific, New Jersey) at 37°C and shaken at 180 rpm for 6 h, after which 5 mL of each culture was inoculated in up to 60 roux flasks (Product code 430641, Corning, UK), each containing 75 mL new sporulation medium (3 g.L^−1^ tryptone peptone (Gibco, 211699), 6 g.L^−1^ bacteriological peptone (Oxoid, LP0037B), yeast extract (Gibco, 212750), Lab Lemco (Oxoid, LP0029B), 1 mL 0.1% wt/wt MnCl_2_:4H_2_O (Thermo Scientific, 04444.2), and 25 g.L^−1^ Difco Bacto agar (Becton, Dickinson, and Company 214010) in MilliQ water, prepared at Dstl and incubated at 37°C (*B. anthracis* Ames) or 28°C (*B. thuringiensis* cry-) for 10 days. Sporulation was confirmed by taking samples from a selection of roux flasks and observing the presence of phase bright spores by oil-immersion phase-contrast microscopy (×1,000 magnification) using a Zeiss Axiolab microscope. If vegetative cells are present, then the incubation time was extended until all cells were sporulated.

Spores were harvested from each Roux flask into 15 mL of sterile distilled water (SDW) and centrifuged at 10,000 × *g* for 15 min at 4°C. After discarding the supernatant, the pellet was re-suspended in sterile distilled water by vortexing in order to wash the spores. This wash step of centrifugation and re-suspension was performed three times. The final washed pellets from each centrifuge pot were re-suspended and combined into approximately 100 mL of SDW, resulting in a concentration of approximately 2 × 10^10^ CFU.mL^−1^. A heat shock assay was performed on 1 mL aliquots of this stock, with and without heat shock at 70°C for 20 min. These were then placed in ice for 20 min to rapidly bring down the temperature and minimize the chance of germination. These were plated on TSA for enumeration and to confirm that there was no difference in spore numbers between the heat-shocked and non-heat-shocked samples.

The Hazard Group 3 *B. anthracis* strains were handled by trained and experienced Dstl staff in specialist facilities at Containment Level 3.

### Surface nebulization

Test coupons were selected to represent porous or non-porous materials commonly found in the urban environment. These were stainless steel 316 grade (20[l] × 20[w] × 1[d] mm; Buy Metal Online, UK), North American Douglas fir wood (20[l] × 20[w] × 6[d] mm; Woodshop Direct, UK), and facing brick (20[l] × 20[w] × 6[d] mm, Wienerberger facing brick; Denton Crofters Medley, UK). These were inoculated with *B. thuringiensis cry-* spores by aerosol deposition to prepare a surface contamination with a nominal target of 7 Log_10_ CFU.cm^−^² of largely dispersed spores as a single layer with some areas of aggregation. This method was reported in a previous study (2) but briefly described here: 5 mL aqueous suspension of spores at 2 × 10^10^ CFU.mL^−1^ was aerosolized for 25 min using a medical nebulizer (Omron NE-C28-E, Omron Healthcare Co., Ltd, Japan) and then allowed to settle for 30 min prior to transfer into individual wells of sterile six-well plates for storage in the dark in an air-conditioned laboratory (20°C) until use.

### Surface spray application and environmental chamber assay

**Fig 1:**
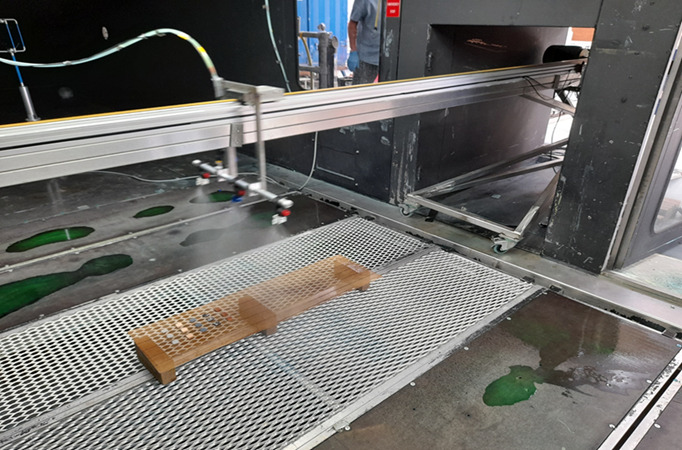
Photograph showing the track-mounted boom with three spray nozzles at 0.25 m spacing and 0.5 m above the inoculated coupons (placed on gauze).

A track sprayer at Silsoe Spray Application Unit (SSAU, Bedford, UK) was used to apply decontaminant spray using full-scale agricultural equipment. Figure 1 shows the track-mounted boom equipped with 3 × Hypro FF110-08 spray nozzles (Hypro EU Ltd, Cambridge, UK) at 0.25 m spacing and positioned 0.5 m above the coupons; this distance is a typical operating height for a vehicle-mounted agricultural boom. The coupons were placed horizontally on a gauze to ensure the sprayed decontaminant did not pool around the coupon and affect the target dose on the surface. The spray lines were operated at 2.0 bar pressure to give a flow rate of 2.6 L.min^−1^ from each nozzle. Target applications of 500 L.ha^−1^, 1,000 L.ha^−1^, and 2,000 L.ha^−1^ were delivered using forward speeds of 3.44 m/s (12.4 kph), 1.72 m/s (6.2 kph), and 0.86 m/s (3.1 kph), respectively.

Following spray application, the test surfaces were weighed, and their difference from their pre-spray weight was calculated. This mass difference was then divided by the liquid density to determine the volume of decontaminant applied to the surface, which was used for dose calculation. The coupons were then transferred to individual wells of a six-well plate and then incubated for 2 h at either 7°C and 94% RH (“mild” condition), or 28°C and 32% RH (“warm” condition), in a pre-conditioned environment chamber (Aralab FITOCLIMA 600 PLH, Portugal). The parameters for the warm condition were selected to be relatively more conducive to evaporative loss than the mild condition.

The time between spraying coupons and placing them into the environment chamber was 10 min. The chamber was preset to the environmental condition, where in addition to the temperature and humidity readout on the front panel of the chamber, these parameters were also monitored using a temperature/humidity data logger (Omega OM-22 multi use PDF, Omega Engineering) placed next to samples inside the chamber. It recorded the cabinet’s internal temperature/humidity every 2 min. Following the placement of coupons into the chamber, the data logger indicated that these parameters returned to the set temperature and humidity within 4 min. The temperature changed by up to ±1.5°C, depending on the preset environmental condition. For humidity, there was no detected change at the time of transition into the cabinet, outside of its ± 2% RH tolerance.

**Fig 2:**
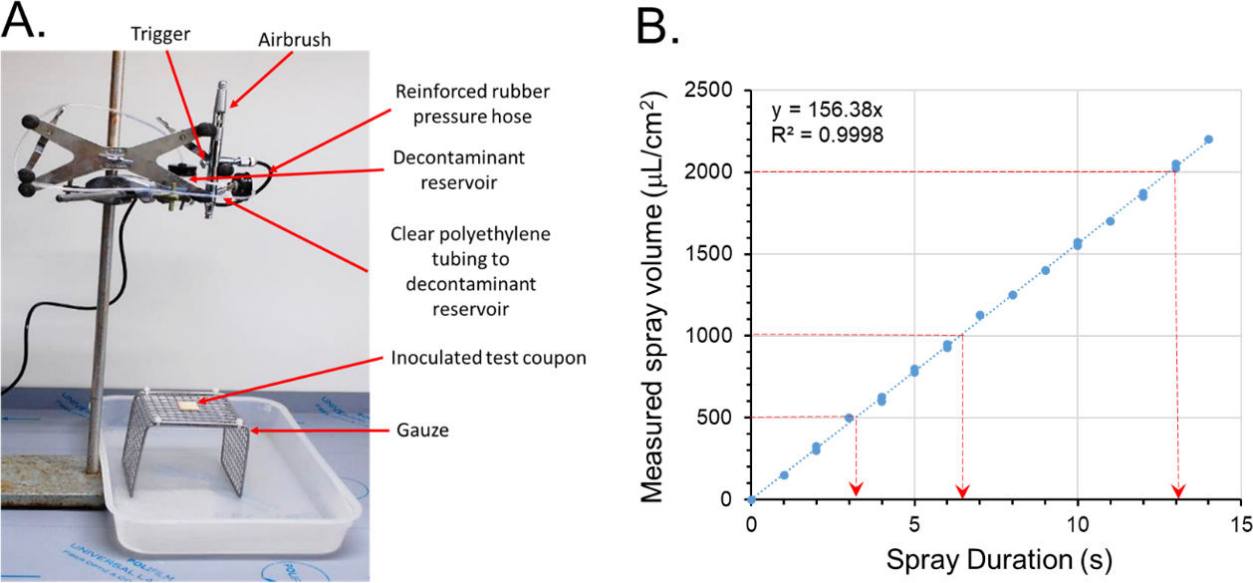
(**A**) Photograph showing the LSSA that provided reproducible sprays to inoculated coupons placed on a metal gauze. (**B**) Calibration graph using water showing the spray duration required to deposit target spray volumes (denoted by the red dashed lines at 500, 1,000, and 2,000 L.ha^−1^) onto coupon surfaces.

Experiments comparing the efficacy of PAA with formaldehyde decontaminants delivered sprays using a previously developed laboratory-scale spray applicator (LSSA) system (2). This was based on a commercial airbrush system (SprayCraft SP50K, UK) as shown in Fig. 2A. Spray delivery onto coupons was calibrated by measuring the quantity on each coupon gravimetrically for different spray durations (Fig. 2B). Reproducible spray volumes of 1.5–22 µL.cm^−2^ (equivalent to 150–2,200 L.ha^−1^) were delivered.

### Spore recovery from coupons, neutralization, and culture

Following the 2 h dwell in the environment chamber, coupons were placed individually into Falcon tubes containing 8 mL neutralizer (1% wt/wt sodium bisulfite) (Product code 243973, Sigma Aldrich, UK) in SDW with 0.05% wt/wt Tween80 (Product code P1754, Sigma Aldrich, UK) so that they were fully immersed; the total quantity of sodium bisulfite was 80,000 µg. Since the highest application was 2,100 L.ha^−1^ of 4.8% wt/wt PAA (see the Results section), the maximum quantity of unreacted decontaminant that could be transferred from an 8.8 cm^2^ surface (largest test coupon, excluding bottom surface) into the neutralizer was 184.8 µL, which contained 8,871 µg PAA. Since, 1 ppm PAA requires 1.37 ppm sodium bisulfite (20), the quantity of sodium bisulfite required for PAA neutralization was 12,153 µg. Therefore, the neutralizer solution was in excess so that it could also neutralize hydrogen peroxide, which existed in greater quantity than PAA by a factor of ~1.53 (determined from the Certificate of Analysis supplied by Airedale Chemical, UK) and required 3.06 ppm sodium bisulfite per 1 ppm of peroxide. Control tests that applied200 µL 5% wt/wt PAA decontaminant into 8 mL sodium bisulfite confirmed successful neutralization with PAA and hydrogen peroxide colorimetric test strips (Quantofix, UK), data not shown.

Following PAA neutralization, 1 g play sand (B&Q, UK) was added to the Falcon tube and then vortexed for 30 s to mobilize spores from the coupon surface and into the sodium bisulfite media. After the sand settled and a 15-min residence time, a dilution series was prepared with sterile distilled water (SDW, −1 to −6). Two streaks of 100 µL aliquots from the appropriate dilution (or neat supernatant) were plated onto one TSA (Product code 11965345, Oxoid, UK,) plate and incubated at 30°C overnight before counting the colony forming units.

For formaldehyde (3% wt/wt) experiments, coupons were placed into Falcon tubes with 8 mL Dey Engley Broth (DEB) neutralizer (Sigma Aldrich, UK, product code D3435; prepared as a 39 g/L solution), which contained 2.5 g/L sodium bisulfite (0.02 g sodium bisulfite in 8 mL DEB). Therefore, the neutralizer was in excess, since the greatest applied volume of formaldehyde solution to the coupon was 1,493 L.ha^−1^, where a maximum of 0.131 mL (0.01493 mL.cm^−2^ × 8.8 cm^2^) containing 0.004 g formaldehyde could be transferred into the neutralizer.

### Surface moisture measurements

Surface moisture measurements were taken over a 2-h period using a Protimeter (Protimeter, Surveymaster 2 Moisture Meter BLD5365) on wood and brick surfaces, following spray application of water (containing the wetting agent: 0.2% wt/wt Synperonic PE L62/L64) at 500, 1,000, or 2,000 L.ha^−1^ and under the mild and warm environmental conditions. These data could not be taken on steel as it was a conductive surface that interfered with the method of measurement.

### Suspension tests

These tests determined the efficacy of PAA formulations, with or without pH 6 adjustment, to inactivate *B. thuringiensis* cry- or *B. anthracis* Ames. An aliquot of 500 µL spores was added to 4.5 mL decontaminant concentrate or water-Synperonic PE L62/:64 (1:1) control to give initial inoculums of 8.22 ± 0.08 Log_10_ CFU.mL^−1^ (*B. thuringiensis* cry-) or 8.59 ± 0.04 Log_10_ CFU.mL^−1^ (*B. anthracis* Ames). The final nominal concentration of the PAA decontaminant (with or without pH adjustment) was 0.5% wt/wt containing 0.2% wt/wt Synperonic PE L62/:64 (1:1) in SDW. The decontaminant that was pH-adjusted contained 0.36% wt/wt sodium hydroxide. Potentiometric titrations confirmed the initial T0 concentration of the pH-adjusted formulation was 0.51% wt/wt PAA. A pipette was used to mix the spores into the decontaminant prior to securing the lid of the Falcon tube. This was then shaken to further induce mixing. The suspension was incubated at 20°C for 5 or 15 min before a 500 µL aliquot was removed and added to 4.5 mL of a 1% wt/wt sodium bisulfite solution for neutralization. A dilution series was prepared and then plated onto TSA for overnight culture and colony counting, as detailed above.

Neutralizer efficacy tests were performed by adding 500 µL decontaminant to 4.5 mL of the neutralizer and then shaking the solution. Following this, 500 µL spore stock (in SDW) was added and incubated for 5 min at 20°C before being sampled and directly plated onto TSA, or preparation of a dilution series prior to plating. Plates were incubated under the conditions stated above.

### Data processing and statistics

For the agricultural track-sprayer experiments, Log_10_ reductions, for a given surface type, were calculated by subtracting the mean post-dwell recoveries of the PAA treated coupons from the mean recoveries of their respective water spray positive controls (See Table 2 in the results section), for each test run.

**TABLE 2:** Control recoveries from inoculated steel, wood, and brick coupons pre- or post-water spray application^*a*^

Control	Surface	Environmental condition	Mean recovery Log_10_ CFU.cm^−2^
Pre-spray (direct recovery)	Steel	Mild	7.25 ± 0.17
		Warm	7.19 ± 0.01
	Wood	Mild	7.61 ± 0.06
		Warm	7.70 ± 0.02
	Brick	Mild	7.33 ± 0.01
		Warm	7.49 ± 0.00
Water spray (2,000 L.ha^−1^)	Steel	Mild	7.21 ± 0.34
		Warm	7.13 ± 0.02
	Wood	Mild	7.49 ± 0.00
		Warm	7.68 ± 0.01
	Brick	Mild	7.31 ± 0.01
		Warm	7.45 ± 0.02

^*a*^ Data shown are the mean of three replicates (±standard error of the mean), except for brick, which was the mean of two replicates.

For experiments that delivered sprays using the LSSA, the Log_10_ reductions, for a given surface type, were calculated by subtracting the mean post-dwell recoveries of the PAA or formaldehyde-treated coupons from the mean recoveries (*n* = 3) of their respective untreated positive controls (i.e., direct recovery of nebulized coupons).

The errors associated with repeat measurements were calculated with the standard deviation of the mean unless otherwise stated. Equality of variance was assessed using Levene’s *F*-test, and data normality was assessed using the Shapiro-Wilk test with OriginPro 2025 SR1 (10.2.0.196, OriginLab Corporation, USA). These were performed prior to the following parametric tests: one-way analysis-of-variance (ANOVA) using the *F*-statistic to test for significant differences between the mean measurements of two or more groups, followed by a *post-hoc* Tukey test for significant differences between the means of subsets of groups. The independent two-samples *t*-test was performed to determine significant differences between the means of two independent sample data sets. Two-way ANOVA was performed using the F-statistic to assess the main effects and interaction effects between two independent variables on the dependent variable. All statistical tests were performed at the 95% CI.

## RESULTS

### Stability of pH-adjusted PAA formulations

**Fig 3:**
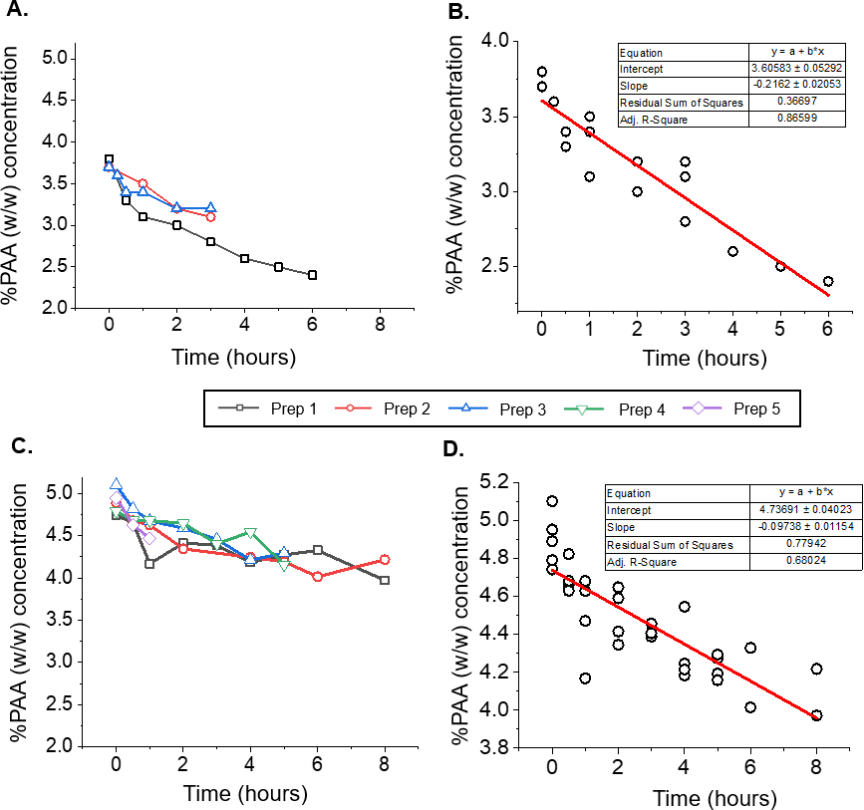
Potentiometric determination of the temporal %PAA (wt/wt) concentration at 21°C for (**A**) three independent preparations of Formulation 1 (without rheology modifier). (**B**) Linear regression model fitted to a scatterplot of the sampling points from Formulation 1, independent of the preparation. (**C**) Five independent preparations of Formulation 2 (with rheology modifier). (**D**) Linear regression model fitted to a scatterplot of the sampling points from Formulation 2, independent of the preparation.

Accurate determination of the initial PAA concentration in each formulation, following pH adjustment, was essential to calculate the applied dose when combined with spray volume data. This enabled correlation with microbiological measurements of spore inactivation in downstream experiments. Figure 3 shows the temporal changes in the %PAA (wt/wt) concentration for Formulations 1 and 2, following three or five independent preparations at 21°C. It is important to note that in-field preparations of the decontaminant would aim not to exceed 30°C, as previous research demonstrated a 4.5 times increase in PAA decomposition between 20°C and 40°C (21). The threshold of 30°C is also the required storage temperature of PAA by the manufacturer (Airedale Chemicals). Moreover, when sodium hydroxide is added to PAA, the temperature of the solution rises due to the exothermic reaction, where this increase is dependent on the rate of addition. Therefore, a decontaminant solution temperature of 21°C ensures adequate separation from the 30°C threshold. This is achieved in practice by starting with a low baseline temperature for PAA to account for a ca. 10°C rise when sodium hydroxide is added.

The measured mean PAA concentrations taken <5 min following preparation (T = 0 h) were 3.7% ± 0.0% and 4.9% ± 0.1% for Formulations 1 and 2, respectively (from an initial nominal dilution of 5% wt/wt).

Although the formulations in this work were used immediately after preparation, in-field use could involve longer periods before the decontaminant is sprayed. To that end, each formulation was sampled at extended time points to determine pot-life. Over 3 h, the %PAA in Formulation 1 had reduced to 3.0%, whereas it was reduced to 4.6% in Formulation 2 during the same period. Linear regression models fitted to a scatterplot of all sampling points (up to 6 or 8 h) and independent of the preparation (Fig. 3C and D) demonstrated a steeper negative slope for Formulation 1 (−0.2162), suggesting a higher reduction in %PAA per unit increase in time, relative to Formulation 2 (−0.09738).

### PAA efficacy on inoculated steel, wood, and brick surfaces under different environmental conditions

Agricultural sprayers were used to apply measured volumes of the formulations to inoculated surfaces. These were then combined with the PAA concentration measurements (described above) and the microbiological kill data presented below (following a 2 h dwell under “mild” or “warm” environmental conditions) to determine dose response.

Prior to these dose-response experiments, control experiments were performed to determine whether the spray application itself resulted in the removal of spores as this can affect assessment of decontamination efficacy on surfaces in subsequent experiments. In Table 2, we show the recoveries from inoculated steel, wood, and brick coupons pre- (no spray, direct recovery) or post-water spray application. Two-way ANOVA was performed on the “mild” data set, which confirmed that there was no significant difference between recoveries pre- or post-water spray across all surfaces (mean 7.4 ± 0.3 Log_10_ CFU.cm^-^²; *P* = 0.92 for interaction between “surface type” and “pre-/post-water spray”). This result was also obtained for the same interaction using the “warm” data set (mean 7.4 ± 0.2 Log_10_ CFU.cm^-^²; *P* = 0.17). Note that the application rate of the applied spray was 2,000 L.ha^−1^, which was the greatest amount applied in the proceeding decontamination efficacy study.

**Fig 4:**
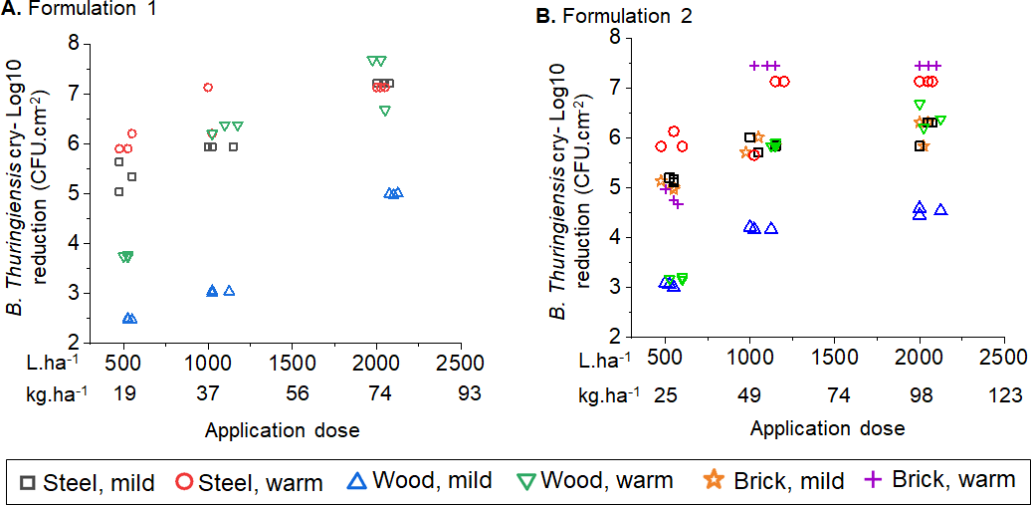
*B. thuringiensis* cry- Log_10_ reduction on steel and wood surfaces following spray application of (**A**) Formulation 1 and (**B**) Formulation 2 at different PAA doses using the agricultural sprayer, and then exposed to either mild (7°C and 94% RH) or warm (28°C and 32% RH) environmental conditions for 2 h. Additional data on inoculated brick were collected following treatment with Formulation 2. The Log_10_ reductions were calculated against recoveries from the water spray controls.

Given that there was no effect of the spray of water on spore removal, we proceeded to collect dose-response data for Formulation 1 and 2 when applied across different surfaces (steel, wood, and brick) and environmental conditions (mild vs. warm). Figure 4 compares the Log_10_ CFU.cm^-^² reduction of *B. thuringiensis* cry- spores on steel and wood coupons when treated with different doses of Formulations 1 and 2 using the agricultural sprayer and then exposed to either mild or warm environmental conditions for 2 h. Additional data were collected from inoculated brick treated with Formulation 2. The dose (kg.ha^−1^) axis was calculated using the measured T = 0 h PAA concentration for the respective formulation (presented above) and the Log_10_ reductions.

**Fig 5:**
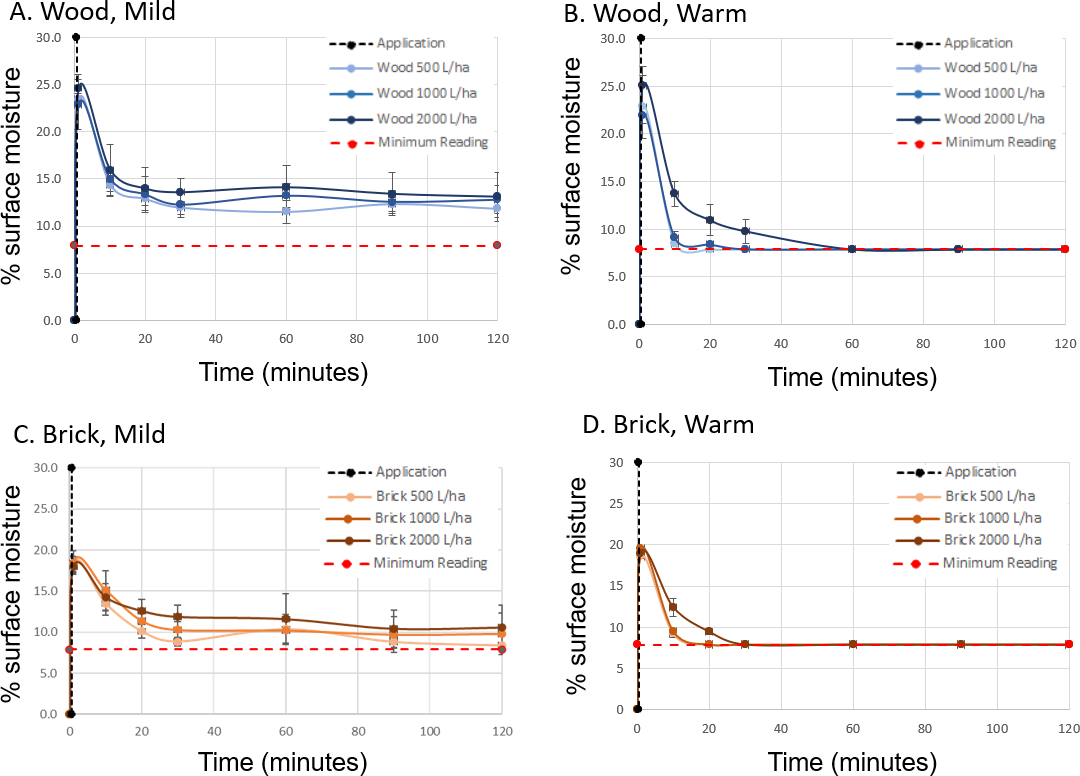
Surface moisture readings on wood (**A and B**) and brick (**C and D**) surfaces following the application of water with 0.2% wt/wt Synperonic PE L62/L64 at 500, 1,000, or 2,000 L.ha^−1^ and then exposed to mild (7°C and 94% RH) or warm (28°C and 32% RH) environmental conditions.

A general trend of increasing spore inactivation with increasing dose was observed in Fig. 4 when treated with either Formulation 1 or 2, for all surfaces and environmental conditions. Another general trend across all surface types and for both formulations was that the level of spore killing achieved at a given nominal PAA dose (19, 37, or 74 kg.ha^−1^) was either comparable between mild and warm conditions or higher under the warm conditions. The latter was unexpected, given that the decontaminant residence time at the surface was anticipated to be lower under the warm conditions. To understand this further, Protimeter moisture measurements were taken, which showed that for both brick and wood, moisture was retained at the surface for longer under the mild condition compared with the warm (Fig. 5). While moisture was detected for up to 120 min under mild conditions using the lowest application volume (500 L.ha^−1^), the same application under warm conditions resulted in no detectable signals at 20 min on wood or brick. At higher application volumes, no detectable signal was obtained at 30 min (brick) or 60 min (wood). These data, together with the Log_10_ reduction trends observed in Fig. 4, and the 10-min interval between spraying and placing the coupons into the chamber, indicated that a large proportion of spore inactivation was achieved within a short period (ca. ≤ 40 min).

Application of Formulation 1 onto impermeable steel at the lowest tested volume 508 ± 38 L.ha^−1^ (*n* = 6) of 3.7% PAA, providing a dose of 19 ± 1 kg.ha^−1^, resulted in Log_10_ reductions of 5.3 ± 0.3 CFU.cm^-^² (mild condition) and 6.0 ± 0.2 CFU.cm^-^² (warm condition) at the 2 h sampling time. Using Formulation 2, there were no significant differences compared to Formulation 1 in Log_10_ reductions measured over the same period and application volume, despite the slightly higher PAA dose of 26 ± 2 kg.ha^−1^ in Formulation 2: 5.2 ± 0.0 CFU.cm^-^² (mild condition; *P* = 0.40) and 5.9 ± 0.2 CFU.cm^-^² (warm condition; *P* = 0.60).

The highest volume of Formulation 1 applied to steel was 2,033 ± 30 L.ha^−1^ (*n* = 6) of 3.7% PAA, which provided a dose of 75 ± 1 kg.ha^−1^. This resulted in no detectable spores observed on the TSA plates under both environmental conditions: a minimum of 7.2 ± 0.3 CFU.cm^-^² (mild condition) and 7.1 ± 0.0 CFU.cm^-^² (warm condition) Log_10_ reduction. While the highest dose of Formulation 2 was applied at 100 ± 2 kg.ha^−1^ (2,042 ± 34 L.ha^−1^ of 4.9% PAA, *n* = 6) and achieved no detectable spores under the warm condition, the mild condition did result in detectable spores with a reduction of 6.2 ± 0.3 Log_10_ CFU.cm^-^². Together, the above data indicated no significant improvement in spore killing using Formulation 2 on horizontal non-porous surfaces, for either environmental condition, despite its slightly higher PAA content.

Application of both formulations onto the absorbent surfaces, wood and brick, resulted in comparable or lower levels of spore killing than what was measured from the steel surface. On porous wood, the lowest tested dose was 19 ± 1 kg.ha^−1^ (525 ± 16 L.ha^−1^ of 3.7% PAA, *n* = 6), which resulted in Log_10_ reductions of 2.5 ± 0.0 Log_10_ CFU.cm^-^² (mild condition) and 3.8 ± 0.0 Log_10_ CFU.cm^-^² (warm condition) at the 2 h sampling time. Unlike the case for steel, application of Formulation 2 (containing the rheology modifier) to the wood surface did show a small but significant improvement in spore killing, where a dose of 27 ± 2 kg.ha^−1^ (550 ± 41 L.ha^−1^ of 4.8% PAA, *n* = 6) at the same volume resulted in 3.1 ± 0.0 Log_10_ CFU.cm^-^² (mild condition; an increase of 0.6 Log10 CFU.cm^-^², *P* = 0.00). However, this improvement was not measured under the warm condition when using the low application dose (3.2 ± 0.0 Log_10_ CFU.cm^-^²; a decrease of 0.6 Log_10_ CFU.cm^-^², *P* = 0.00). The small reduction in efficacy when applying Formulation 2 (compared with Formulation 1) was also measured using the high dose application of 76 ± 2 kg.ha^−1^ (2,058 ± 54 L.ha^−1^ of 4.8% PAA, *n* = 6): reductions of 5.0 ± 0.0 Log_10_ CFU.cm^-^² with Formulation 1 and 4.5 ± 0.1 Log_10_ CFU.cm^-^² with Formulation 2 were measured for the mild condition; reductions of 7.3 ± 0.6 Log_10_ CFU.cm^-^² and 6.4 ± 0.2 Log_10_ CFU.cm^-^² with Formulations 1 and 2, respectively, were measured under the warm condition.

Using Formulation 2, spore killing on the brick surface at each applied volume and environmental condition was higher than that achieved on wood employing the same parameters. Moreover, under the warm conditions, no detectable spores were measured using the high dose of 100 ± 2 kg.ha^−1^ (2,038 ± 38 L.ha^−1^ of 4.8% PAA, *n* = 6), which indicated a minimum of 7.5 ± 0.0 CFU.cm^-^² Log_10_ reduction.

### Assessment of sporicidal efficacy between PAA and formaldehyde

In this section, we determined whether PAA applied at comparable concentrations and volumes exhibited sporicidal efficacy against *B. thuringiensis* cry- on wood surfaces that was not significantly different from the efficacy achieved with formaldehyde.

**Fig 6:**
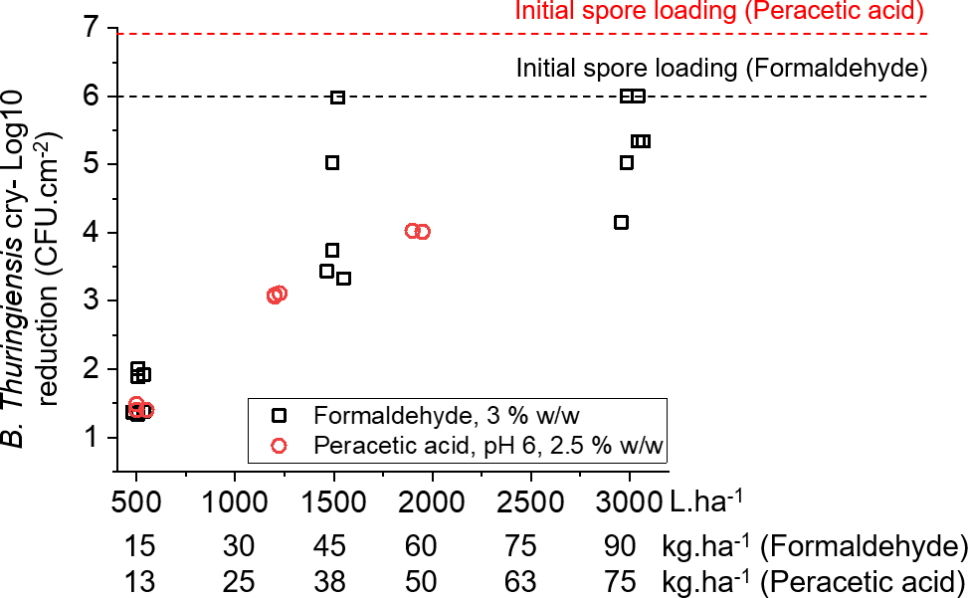
*B. thuringiensis* cry- Log_10_ CFU.cm^−2^ reduction on wood surface following treatment (LSSA spray) with 3% wt/wt formaldehyde or 2.5% wt/wt PAA and a 2 h contact time at 21°C.

The sporicidal efficacy of 3.0% wt/wt formaldehyde to inactivate *B. thuringiensis* cry- on wood was compared with PAA for a comparable concentration and application volume (Fig. 6). Although the initial starting quantity of PAA was a nominal dilution of 3.0% wt/wt (also included 0.2% Synperonic PE L62/L64), the measured PAA concentration was 2.5% ± 0.0% wt/wt upon adjustment to pH 6.0. Consequently, the dose axis scale in Fig. 6 was calculated using the measured concentration. The applied volumes were in the nominal range of 500–3,000 L.ha^−1^. Note that the initial spore loadings on the wood coupons between the were 5.97 ± 0.0 Log_10_ CFU.cm^-^² and 6.89 ± 0.0 Log_10_ CFU.cm^-^² for the formaldehyde and PAA data sets, respectively.

The data showed a reduction of 1.6 ± 0.3 Log_10_ CFU.cm^-^² (*n* = 9) upon treatment with formaldehyde using 507 ± 20 L.ha^−1^ (dose 15 ± 0.3 kg.ha^−1^). A comparable volume (517 ± 29 L.ha^−1^) and dose of PAA (13 ± 0 kg.ha^−1^) resulted in a reduction of 1.4 ± 0.1 Log_10_ CFU.cm^-^² (*n* = 3), which was not significantly different from that achieved with formaldehyde (two-samples *t*-test, *P* = 0.46). There was also no significant difference in efficacy between formaldehyde and PAA when higher doses of 45 ± 1 kg.ha^−1^ (*n* = 6) and 39 ± 1 kg.ha^−1^ (*n* = 6) were applied, respectively; reductions were 4.4 ± 1.1 with formaldehyde and 3.6 ± 0.5 using PAA (two-samples *t*-test*, P* = 0.10).

### Comparative assessment of the sporicidal efficacy of PAA formulations against virulent *B. anthracis* Ames vs. *B. thuringiensis* cry-

Differences in the susceptibility of *B. thuringiensis* cry- and *B. anthracis* Ames to PAA-based decontaminants, with and without pH adjustment, were assessed using suspension tests. This analysis was undertaken to determine how efficacy data generated with the Hazard Group 1 (HG1) strain can be extrapolated to the virulent Hazard Group 3 (HG3) strain, recognizing that a wider range of test conditions (including surface decontamination and varied environmental parameters) can be more readily evaluated with HG1.

The effectiveness of the neutralizer was determined prior to these tests, where Table 3 shows no significant changes in viable spore counts of *B. thuringiensis* cry- when added to 4.5 mL neutralizer medium, pre-spiked with 0.5 mL decontaminant (0.5% PAA, *P* = 0.15). The experiment was performed using *B. anthracis* Ames, where a small but statistically significant drop in viable count was measured (mean difference of 0.3 Log_10_ CFU/mL for each formulation [*P* = 0.00]).

**TABLE 3:** Mean (±SEM) *B. anthracis* Ames and *B. thuringiensis* cry- spore Log_10_ counts following control tests for neutralizer efficacy^*a*^

Spore	Treatment	Mean spore count (Log_10_ CFU.mL^−1^)
*B. anthracis* Ames	Viable count	8.22 ± 0.08
	Neutralizer efficacy (PAA, 0.5% wt/wt)	7.94 ± 0.02
	Neutralizer efficacy (PAA, 0.5% wt/wt, pH 6)	7.92 ± 0.01
*B. thuringiensis* cry-	Viable count	8.59 ± 0.04
	Neutralizer efficacy (PAA, 0.5% wt/wt)	8.59 ± 0.03

^*a*^ Data shown are the mean of three replicates (±SEM), except for the viable counts, which are five (*B. anthracis* Ames) or four (*B. thuringiensis* cry-).

**Fig 7:**
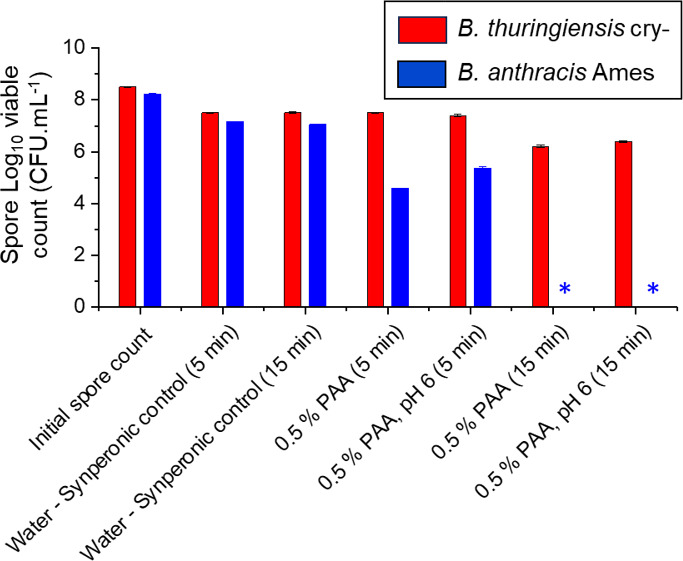
Graph to compare initial mean (±standard error of the mean) *B. anthracis* Ames and *B. thuringiensis* cry- spore Log10 counts with those following exposure to water-Synperonic control medium or decontaminants (0.5% PAA or pH 6 adjusted 0.5% PAA) for 5 or 15-min contact times. The asterisk indicates decontamination parameters where no CFU were observed on TSA plates for *B. anthracis* Ames. The number of replicates was *N* = 3 per test variable.

Exposing *B. thuringiensis* cry- spores to the water-Synperonic control (decontaminant in the absence of PAA) for 5 or 15 min (and then neutralizer for 5 min) resulted in mean Log_10_ reductions of 1.04 ± 0.02 and 1.03 ± 0.03 CFU.mL^−1^, respectively (Fig. 7). A comparable result was obtained for *B. anthracis* Ames, which showed a mean Log_10_ reduction of 1.10 ± 0.03 CFU.mL^−1^ (5-min treatment) or 1.17 ± 0.10 CFU.mL^−1^ (15-min treatment).

Treating *B. anthracis* Ames (8.22 ± 0.08 Log_10_ CFU.mL^−1^) with 0.5% wt/wt PAA (pH-adjusted or non-adjusted) for 15 min resulted in no detectable spores on the TSA plate. However, spores of *B. thuringiensis* cry- were countable and demonstrated Log_10_ reductions of 2.43 ± 0.05 CFU.mL^−1^ (15 min exposure to 0.5% wt/wt PAA) or 2.23 ± 0.03 Log10 CFU.mL^−1^ (15 min exposure to 0.5% wt/wt PAA, pH 6). The reduced susceptibility of *B. thuringiensis* cry- spores to PAA was also observed at the shorter contact time of 5 min: Log_10_ reductions of 1.17 ± 0.05 CFU.mL^−1^ for *B. thuringiensis* cry- upon exposure to pH 6-adjusted PAA vs*.* 2.87 ± 0.06 CFU.mL^−1^ for *B. anthracis* Ames under the same conditions; Log_10_ reductions of 1.06 ± 0.02 CFU.mL^−1^ for *B. thuringiensis* cry- upon exposure to PAA (no pH adjustment) vs*.* 3.63 ± 0.01 CFU.mL^−1^ for *B. anthracis* Ames under the same conditions.

These suspension tests were also used to evaluate the effect of adding sodium hydroxide to the PAA formulation on spore viability. Independent two-samples *t*-tests determined no significant difference between *B. thuringiensis* cry- spore Log_10_ reductions treated for 5 min with 0.5% wt/wt PAA, with or without pH adjustment (*P* = 0.40). A small but significant reduction in spore killing was obtained when *B. thuringiensis* cry- spores were treated with the pH-adjusted PAA, compared to PAA without pH adjustment using an extended contact time of 15 min (*P* = 0.03; mean difference of 0.2 Log_10_ CFU.mL^−1^). Moreover, the trend was also measured using *B. anthracis* Ames, where treatment of spores for 5 min with pH-adjusted PAA resulted in lower kill than PAA without pH adjustment (*P* = 0.00; mean difference of 0.8 Log_10_ CFU.mL^−1^).

## DISCUSSION

Planning an effective, rapid, and logistically viable decontamination strategy over a wide contaminated urban area is critically dependent on dose-response information for formulations that can be prepared in large quantities and are compatible with spray delivery systems. To that end, we adjusted PAA to a weakly acidic pH to improve its material compatibility. However, the reactivity of PAA is highly dependent on its speciation, where at pH ≥ 5.5 the PAA anion forms, which can attack the neutral PAA molecule, resulting in its decomposition (22). Consequently, the reduction potential of PAA decreases from 1.75 to 1.01 V as the pH increases from 0 to 14 (23). Conforming to the expected decomposition upon pH adjustment, we measured an initial drop from a nominal dilution of 5% wt/wt to 3.7% ± 0.0% wt/wt for Formulation 1 (Fig. 3A and B). However, this drop was not observed in Formulation 2, which contained the rheology modifier (Kelzan AP-AS) with an initial PAA concentration of 4.9% ± 0.1% wt/wt (Fig. 3C and D). The improved stability may be attributed to diffusion-limited reactions between the PAA anion and its neutral molecule because of Kelzan AP-AS (a polysaccharide xanthan gum) imparting increased viscosity as it absorbs water.

Diffusion kinetics may also play an important role in the sporicidal efficacy of the PAA formulations, as discussed in the following text. Surface moisture retention data confirmed that while evaporative loss was higher under the “warm” environmental condition compared to the “mild” condition (Fig. 5A through D), the level of spore killing was greater under this warm condition on all surface types tested (steel, brick, and wood). These data indicated that a large proportion of the spore killing was achieved within a short period (ca. ≤ 40 min; this includes the 10-min interval between spraying and placing the coupons into the chamber), where it was possible that the higher temperature under the warm condition could have increased the rate of oxidation reactions and/or PAA molecular mobility through the spore’s exosporium coating to disrupt proteins and lipids in its inner membrane. Interestingly, the sporicidal efficacy of Formulation 2 was generally lower than that of Formulation 1 on absorbent wood for a given application volume and environmental condition, despite its higher dose. This result may be due to diffusion-limited mass transport of PAA molecules to the spores in the presence of the rheology modifier in Formulation 2.

The higher level of *B. thuringiensis* cry- spore killing observed on porous brick vs*.* porous wood using Formulation 2 (Fig. 4B) is likely weighted by their differences in chemical composition (organic vs. inorganic) rather than their porosity. This is evident upon contrasting the spore killing data with the moisture retention data in Fig. 6, which demonstrated either comparable levels over the 2-h period under the mild condition or a shorter retention time on the brick under the warm condition. Oxidative reactions between PAA and the lignin or cellulose components of wood likely dominate the availability of PAA at the surface, compared with the minimal or no chemical reactions between PAA and the brick surface.

We compared dose-response data for peracetic acid (pH 6 but no rheology modifier) with formaldehyde applied to *B. thuringiensis* cry- inoculated wood using comparable volumes (ca. 500 L.ha^−1^ and 1,500 L.ha^−1^), active substance concentration (ca. 3% wt/wt), contact time (2 h), and environmental condition (21°C). The data in Fig. 7 showed similar levels of spore killing at each dose or applied volume despite the different modes of inactivation by each decontaminant. Formaldehyde is known to inactivate spores through binding to nucleic acid, causing DNA cross-linking and fragmentation (24), unlike PAA, which induces oxidative damage to biomolecules.

Overall, this work reported the performance of a scalable and effective PAA-based decontaminant that enables remediation planners to formulate logistically viable strategies for wide area decontamination of *B. anthracis* based on application parameters (L.ha^−1^), surface type (porous/non-porous), and environmental conditions. Formulation 1 consisted of peracetic acid (PAA) adjusted to pH 6 at a concentration of 3.7% wt/wt (30 g·L⁻¹). When applied at a rate of 2,046 ± 43 L·ha⁻¹ (equivalent to 0.2 L·m⁻²), it delivered a dosage of 76 kg·ha⁻¹. Across the tested environmental conditions, this treatment achieved a reduction of ≥ 5 Log_10_ CFU·cm⁻² (from an initial level of 7 Log_10_ CFU·cm⁻²) within a 2-h sampling period on both porous and non-porous surfaces. Under the same parameters, lower inactivation was measured on these surfaces with Formulation 2 (≥4.6 Log_10_ CFU·cm⁻²), despite a higher dose (98 kg·ha⁻¹) from its initial PAA concentration.

In a previous study, peracetyl borate (PES solid) was utilized as a precursor to generate PAA. A 5% wt/wt solution (50 g·L⁻¹) was applied at a volume of 2 mL to 2 × 2 cm test coupons inoculated with *Bacillus anthracis* Ames, *B. anthracis* Sterne, or *B. thuringiensis*, corresponding to an application rate of 50,000 L·ha⁻¹ (25). This treatment achieved either complete spore inactivation or reduced survival to below 1 Log_10_ (from an initial load of 7 Log_10_ spores per coupon, equivalent to 6.4 Log_10_ CFU.cm^−2^) across various representative porous and non-porous aircraft surface materials within a 15-min contact period. In contrast, the findings from our study demonstrated that comparable levels of spore reduction (≥5 Log_10_ CFU·cm⁻²) could be achieved using significantly lower application volumes (2,046 ± 43 L·ha⁻¹), thereby offering a more practical and resource-efficient approach for large-scale decontamination across surface types and environmental conditions.

However, it is important to consider that the findings presented in this study, along with those by Buhr et al. (25), were obtained using clean surfaces without urban grime. Additional research is needed to assess how such surface contaminants may reduce the availability or efficacy of the active substance.

Other important considerations in future work include the need for full-scale field trials to fully integrate all variables likely to be encountered in a large-scale remediation effort, which can impact efficacy. As well as a wider range of surface types of varying porosity and surface phobicity, this can include decontamination of complex surfaces such as turf (sward and soil), trees, and shrubs. Previous research by the EPA contaminated these complex surfaces with spores of *Bacillus atrophaeus* var. *globigii (Bg)* (another surrogate for *B. anthracis*) to subsequently assess the efficacy of PAA delivered using an agricultural boom sprayer (turf) or handheld spray gun (trees and shrubs) (26). These workers applied 0.5% wt/wt PAA at 36 gal.1,000 ft^−2^ (14,650 L.ha^−1^) to turf, or 50 gal.1,000 ft^−2^ (20,350 L.ha^−1^) to trees and shrubs, which delivered doses of 73 kg.ha^−1^ and 101 kg.ha^−1^, respectively. Although the applied volumes were approximately 10 times larger than those used in our work, the doses still fell within our study’s range and produced between 3 and 5 Log_10_ reductions across the matrices. It is noteworthy to mention that despite the large volumes applied, the authors noted difficulty in achieving uniform surface coverage across the trees and shrubs.

Comparing *B. thuringiensis* cry- spore inactivation with *B. anthracis* Ames for the same PAA dose (with or without pH adjustment) was performed with the view to understanding how data from the HG1 strain could be used to estimate decontamination performance on virulent *B. anthracis* strains and strengthen advice in the event of an operation. The results of Fig. 7 confirmed that a decontamination capability developed using *B. thuringiensis* cry- spores strongly suggests that it will achieve at least the measured level of hazard reduction when applying the same treatment conditions to virulent *B. anthracis* spores or greater efficacy. In a similar context, it was reported that *B. anthracis* Ames had greater susceptibility to inactivation by formaldehyde treatment than to the HG1 surrogate strain *B. atrophaeus* in soil samples (27).

The suspension test data (Fig. 7) also demonstrated that spore counts of *B. thuringiensis* cry- or *B. anthracis* Ames, following treatment with pH-adjusted PAA, were either not significantly different from those obtained without pH adjustment or showed a slight reduction in sporicidal activity. These suspension tests used 0.5% wt/wt PAA-adjusted with 0.4% wt/wt sodium hydroxide, which corresponded to a sodium hydroxide-to-PAA mass ratio of 0.8 and a molar ratio of 1.6. These ratios were similar to those for Formulation 1 (sodium hydroxide: PAA mass ratio of 1.2 and molar ratio of 2.3) and Formulation 2 (sodium hydroxide: PAA mass ratio of 0.8 and molar ratio of 1.4). Collectively, these findings, together with the PAA degradation profiles in Fig. 3, demonstrated that sodium hydroxide did not act as a biocidal component in the formulation and, in fact, reduced the efficacy of PAA by promoting the formation of the PAA anion and accelerating its decomposition. This study demonstrated that pH-adjusted PAA formulations can deliver rapid and effective spore inactivation at application volumes of PAA far lower than previously reported, offering a practical solution for large-scale decontamination. This was achieved under a range of environmental conditions and on different surface types, although further evaluation in the presence of urban grime is warranted to confirm performance. It is important to note that although adjustment of PAA to neutral pH may improve material compatibility (18), suspension test data demonstrated that this pH adjustment did not improve sporicidal efficacy. Finally, we contribute to disinfectant formulation design by showing that while inclusion of a rheology modifier to enhance surface retention on porous surfaces may improve the stability of PAA in pH-adjusted formulation, it can also reduce microbial inactivation due to diffusion limitations. Consequently, its application to the nebulized, porous, horizontal surfaces in this study did not provide a net benefit on sporicidal efficacy.

## Data Availability

The data underlying this article will be shared on reasonable request to the corresponding author.
